# On the Track of Long-Range Electron Transfer in B-Type Dye-Decolorizing Peroxidases: Identification of a Tyrosyl Radical by Computational Prediction and Electron Paramagnetic Resonance Spectroscopy

**DOI:** 10.1021/acs.biochem.1c00129

**Published:** 2021-03-30

**Authors:** Kevin Nys, Paul Georg Furtmüller, Christian Obinger, Sabine Van Doorslaer, Vera Pfanzagl

**Affiliations:** † BIMEF Laboratory, Department of Chemistry, University of Antwerp, 2610 Antwerp, Belgium; ‡ Department of Chemistry, Institute of Biochemistry, BOKU-University of Natural Resources and Life Sciences, 1190 Vienna, Austria

## Abstract

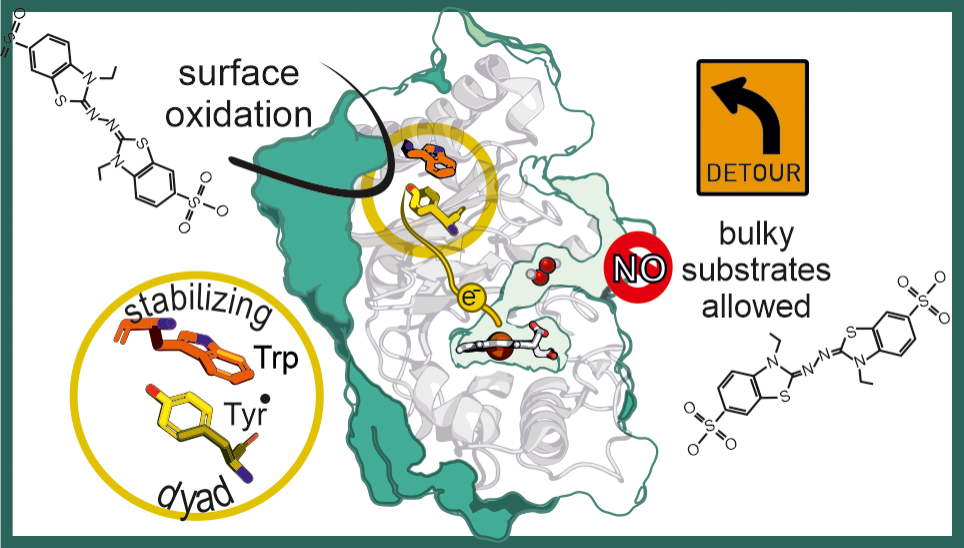

The catalytic activity of dye-decolorizing peroxidases (DyPs) toward bulky substrates, including anthraquinone dyes, phenolic lignin model compounds, or 2,2′-azino-bis(3-ethylbenzothiazoline-6-sulfonic acid) (ABTS), is in strong contrast to their sterically restrictive active site. In two of the three known subfamilies (A- and C/D-type DyPs), catalytic protein radicals at surface-exposed sites, which are connected to the heme cofactor by electron transfer path(s), have been identified. So far in B-type DyPs, there has been no evidence for protein radical formation after activation by hydrogen peroxide. Interestingly, B-type *Klebsiella pneumoniae* dye-decolorizing peroxidase (*Kp*DyP) displays a persistent organic radical in the resting state composed of two species that can be distinguished by W-band electron spin echo electron paramagnetic resonance (EPR) spectroscopy. Here, on the basis of a comprehensive mutational and EPR study of computationally predicted tyrosine and tryptophan variants of *Kp*DyP, we demonstrate the formation of tyrosyl radicals (Y247 and Y92) and a radical-stabilizing Y-W dyad between Y247 and W18 in *Kp*DyP, which are unique to enterobacterial B-type DyPs. Y247 is connected to Y92 by a hydrogen bonding network, is solvent accessible in simulations, and is involved in ABTS oxidation. This suggests the existence of long-range electron path(s) in B-type DyPs. The mechanistic and physiological relevance of the reaction mechanism of B-type DyPs is discussed.

Heme peroxidases mediate the peroxide-dependent oxidation of numerous inorganic or organic molecules, ranging from anions (e.g., halides or thiocyanate), cations (e.g., manganese ions), and organic compounds (e.g., phenols and ascorbate) to proteins (cytochrome *c*). The redox cofactor (heme *b* or posttranslationally modified heme) is located in the interior of the protein and can be accessed by channel(s) for H_2_O_2_ and electron donors.^1^ Typically, the substrate is oxidized at the upper (distal) side of the heme cavity or the heme edge.^2,3^ For oxidation of larger substrates or in peroxidases with restrictive access channels, electron transfer paths from remote aromatic residues to the heme cofactor have been described.^4−8^

In general, heme peroxidases follow the so-called Polous and Kraut mechanism (Figure 1D).^1,9^ Hydrogen peroxide mediates the oxidation of the ferric resting state to Compound I (*k*_1_) [oxoiron(IV) porphyrin π-cation radical].^9^ The reduction of Compound I may proceed either directly in a two-electron reaction (*k*_2_) (e.g., mediated by halides or thiocyanate) or through two consecutive one-electron reduction reactions via a Compound II [oxoiron(IV)] (*k*_3_ and *k*_4_).^1,9−11^ Typically, during the reduction of Compound I, the porphyrin π-cation radical is quenched by substrate-derived electrons. However, quenching of the porphyryl radical can also occur by unspecific or specific internal electron transfer, thereby generating a so-called Compound I*, which exhibits a Compound II-like ultraviolet–visible (UV–vis) spectrum but is electronically a distinct redox state [oxoiron(IV) protein radical] that can be identified by electron paramagnetic resonance (EPR) spectroscopy. In the case of specific internal electron transfer, the resulting protein-based radical at the protein surface can be the site of entry for electrons from bulky (physiological) substrates. In principle, two electrons are necessary for reduction of Compound I* to the ferric resting state (Figure 1D). In addition to allowing the access of bulkier substrates, solvent-exposed binding and oxidation sites allow altered specificity and selectivity compared to direct oxidation at the heme cofactor. Electron entry sites and long-range electron transport (LRET) paths in oxidoreductases are often highly conserved.

**Figure 1:**
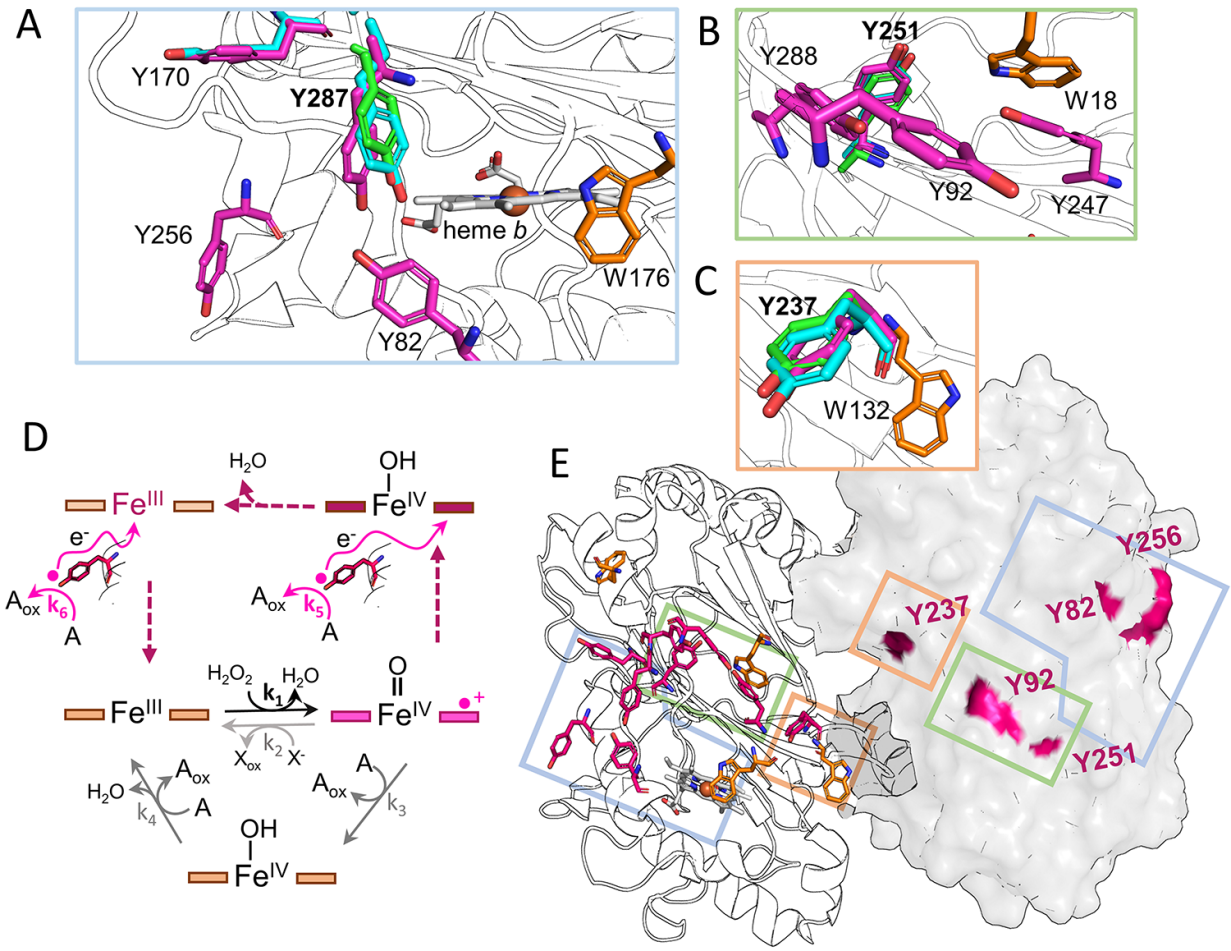
Tyrosine and tryptophan residues in the dye-decolorizing peroxidase from *K. pneumoniae* [*Kp*DyP, Protein Data Bank (PDB) entry 6FKS] form an aromatic girdle in the interface between the dimeric ferredoxin fold. (A–C) Detailed depiction of the aromatic girdle containing all tyrosine and tryptophan residues corresponding to boxes in panel E. Structurally conserved tyrosine residues from aligned structures of D-type *A. auricula-judae* DyP [Y433 (A), Y362 (B), and Y337 (C) (PDB entry 4W7J)] and A-type *Escherichia coli* EfeB [Y269 and Y402 (A), Y352 (B), and Y368 (C) (PDB entry 3O72)] are colored green and cyan, respectively. Heme *b* is colored gray. (D) Graphic representation of the possible intermediates of heme peroxidases formed during the peroxidase cycle. The individual redox states are colored pink, and pink arrows indicate electron transfer from the proteinaceous electron source (tyrosyl in stick representation) to the heme cofactor. Arrows with solid line indicate redox reactions *k*_1_–*k*_6_, and dashed arrows indicate the direction of the reaction cycle only. The porphyrin ring is shown as a box, and the heme iron with the respective oxidation state and ligation. One-electron donors are denoted as A, and two-electron donors as X^–^. The respective oxidation products are A_ox_ and X_ox_. (E) Structure of the *Kp*DyP dimer showing all tyrosine (magenta) and tryptophan (orange) residues in chain A. The backbone is shown as a cartoon representation. Chain B is shown as a surface representation with the surface corresponding to tyrosine residues colored magenta.

One of the best studied examples of a catalytic Compound I* is yeast cytochrome *c* peroxidase (C*c*P). C*c*P activity requires formation of a Trp cation radical (W191^•^^+^) for oxidation of cytochrome *c*.^12^ In *Mycobacterium tuberculosis* catalase-peroxidase, formation of a radical at Y229 of the unique Met-Tyr-Trp adduct is required for its (pseudo-)*catalatic* activity.^13^ It long eluded identification due to its short half-life and subsequent formation of additional radical sites with questionable biological significance.^13−15^ More recently, a tyrosyl radical was shown to play a central role during conversion of coproheme to heme *b* in coproheme decarboxylases (ChdC).^16−18^ Crucially, the radical sites described above are located on highly conserved, catalytically active residues within the protein core and in the proximity of the heme cofactor.

LRET can be directed (specific) or occur spontaneously. Spontaneous (unspecific) electron tunnelling from distant, nonconserved sites was observed in human peroxidases in the absence of substrates.^19−22^ An example of LRET for targeted substrate oxidation on the protein surface is found in lignin peroxidases, where a conserved neutral tryptophanyl (e.g., W171 in *Phanerochaete chrysosporium*(5)) or a tyrosyl radical (e.g., Y181 in *Trametopsis cervina*(23)) promotes the oxidation of veratryl alcohol. In recent years, dye-decolorizing peroxidases (DyPs) have also been shown to harbor amino acid radical sites.^6,8,24,25^ DyPs have attracted interest due to their activity toward anthraquinone dyes and phenolic lignin model compounds.^26^ The DyP family is composed of three phylogenetically, structurally, and biochemically distinct subfamilies (types A, B, and C/D). They share two core features, namely, a dimeric ferredoxin-like fold with one heme *b* cofactor in the C-terminal domain (Figure 1E) and a conserved catalytic Asp-Arg pair, which mediates the formation of Compound I.^27^ While in other peroxidases substrate binding sites at the heme γ- or δ-meso bridges allow direct electron transfer to Compound I (*k*_2_) and Compound II (*k*_3_), the accessibility of the heme *b* cofactor is limited in all DyPs by a narrow channel locked in an extensive hydrogen bonding network.^28^ In B-type *Klebsiella pneumoniae* dye-decolorizing peroxidase (*Kp*DyP), this channel has a bottleneck radius of < 3 Å, effectively blocking access for substrates larger than H_2_O_2_.

Interestingly, the three DyP subfamilies behave differently in view of Compound I stability and decay. In B-type DyPs, Compound I is unusually stable and unreactive toward most reported substrates.^11,29−31^ The formation of Compound I has been demonstrated by UV–vis spectroscopy in B-type DyPs from *Rhodococcus jostii* RHA1,^32^*Enterobacter lignolyticus*,^30^ and *Pseudomonas putida*.^33^ The presence of a porphyrin radical has first been shown in B-type *Kp*DyP^11^ and A-type DyP from *Streptomyces lividans*.^24^ In A- and C/D-type DyPs, Compound I rapidly decays to Compound I*.^8,25^ Tyrosine and tryptophan residues are omnipresent in all DyPs, and several LRET paths and radical sites in surface-exposed aromatic residues have been described.^34,35^ C/D-type DyP from the wood-degrading fungus *Auricularia auricula-judae* (*Aau*DyP) has been shown to form mixed radical species composed of a neutral tryptophan radical (W377), which is the main catalytic site for oxidation of Reactive Blue 19 (RB19), and secondary radical sites at Y337 (i.e., the dominating species in multifrequency EPR spectra) and Y147, which appear to be unrelated to catalytic turnover of RB19, ABTS, and other substrates.^6,7^ Interestingly, Y337 is structurally conserved in all DyP types and redox-active in *Sl*DyP from *S. lividans* (Y374)^24^ and *Thermomonospora curvata* (*Tc*DyP) (Y332).^8^ Studies in *Sl*DyP identified Y374 to be an oxidation site for ABTS.

B-Type DyPs have been shown to catalyze the oxidation of bulky substrates such as RB19, ABTS, and even phenolic lignin model compounds. Their narrow access channel(s) also suggests the requirement for a solvent-exposed oxidation site. However, so far no radicals apart from the Compound I porphyrin radical were observed in B-type DyPs upon reaction with H_2_O_2_. Surprisingly, wild-type *Kp*DyP displays a persistent organic radical in the resting state without prior addition of H_2_O_2_, albeit it is estimated to be present in only 1% of the total protein.^11^ The origin of this radical and localization is difficult to judge, as *Kp*DyP contains nine tyrosine and four tryptophan residues per subunit with five tyrosines being solvent-exposed (Figure 1A–C,E), including the structurally conserved Tyr described above (Y237 in *Kp*DyP). We hypothesize that the organic radical must originate from the peroxidase cycle, as variants with strongly reduced efficiency in the formation of Compound I (D143A and R232A) did not contain this radical.^9^ Here we present a computational approach of LRET prediction in *Kp*DyP by molecular dynamics (MD) simulation combined with site-directed mutagenesis (Y18F, Y82F, Y92F, Y237F, Y247F, W176F, W18F/Y92F, and Y92F/Y247F) and multifrequency electron paramagnetic resonance spectroscopies to elucidate the localization and origin of the “resting-state” radical. We identify a radical site at Y247, exclusively conserved in enterobacterial B-type DyPs, which is stabilized by a Y247-W18 dyad and is involved in substrate oxidation. A reaction mechanism is proposed.

## Materials and Methods

### Site-Directed Mutagenesis and Expression

Expression of wild-type *Kp*DyP (Uniprot accession number A0A0W8ATM9) was performed as described by Pfanzagl et al.^11^ The variants were created by site-directed mutagenesis using the quick change lightning kit (Agilent Technologies, Santa Clara, CA) according to the manufacturer’s description and primers 3′-tgccgtaccaaacggcaagctctggcg-5′ and 3′-cgccagagcttgccgtttggtacggca-5′ (Y237F), 3′-cagtaggcgcagaagaacagaccgtgggtac-5′ and 3′-gtacccacggtctgttcttctgcgcctactg-5′ (Y247F), and 3′-ccttcagattggcttcaatgaaaattgctgcacgacaatgttc-5′ and 3′-gaacattgtcgtgcagcaattttcattgaagccaatctgaagg-5′ (W18F) with the WT plasmid as the template and primers 3′-gaatcagcaaatcaaactgggtgctcggtgccag-5′ and 3′-ctggcaccgagcacccagtttgatttgctgattc-5′ (Y92F) with the respective variant plasmids as the template for the W18F/Y92F and Y92F/Y247F double variants.

The variants were heterologously expressed in *E. coli* BL21 (DE3) Tuner (Merck/Novagen, Darmstadt, Germany) in Luria broth supplemented with ampicillin. Cells were incubated at 37 °C and 180 rpm until the *A*_600_ reached 0.8, and then the cultivation temperature was decreased to 16 °C. Protein expression was induced after 1 h using 0.5 mM isopropyl β-d-thiogalactopyranoside (final concentration), and cells were harvested by centrifugation (4500 rpm, 25 min, 4 °C) after having been grown for 16 h at 16 °C and stored at −30 °C.

Protein purification was performed as described by Pfanzagl et al., with an alteration in the reconstitution with hemin.^11^ Reconstitution was performed after affinity purification by addition of a 2-fold excess of hemin and a 30 min incubation at room temperature. All samples were filtered through a 22 μm filter and further purified by size exclusion chromatography using a HiLoad 16/60 Superdex 200 prep grade column (GE Healthcare), pre-equilibrated with 50 mM phosphate buffer (pH 7.0) as described previously. All fractions with RZ values of >2 were concentrated and stored at −80 °C.

The genes encoding YfeX (*Ec*DyP) were amplified from genomic DNA using primers 5′-aaaaggatccatgtctcaggttcagagtggc-3′ and 5′-aaactcgagttacagcgccatcaacttgtcca-3′ and cloned into plasmid pGEX-6P1 in frame with the GST tag using sticky end ligation and restriction sites BamHI and XhoI. Expression and purification were performed as described for wild-type *Kp*DyP.

### Steady-State Kinetics

The kinetic parameters for the oxidation of ABTS were determined by following the absorbance change at 414 nm (ε_414_ = 36000 M^–1^ cm^–1^) for 60 s at room temperature using a stirred cuvette and a Cary 60 spectrophotometer (Agilent Technologies). The absorbance change (Δ*A*) was determined using 1 mL of 50 mM phosphate-citrate buffer (pH 5.0) with 10–80 and 20–200 μM (Y247F and Y92F/Y247F, respectively) ABTS, 40 μM H_2_O_2_, and 100 nM enzyme. All reactions were carried out in triplicate. Michaelis–Menten parameters were determined using nonlinear least-squares fitting with Sigma plot 14.

### Pre-Steady-State Kinetics

Pre-steady-state spectroscopic changes induced by addition of H_2_O_2_ were measured using a SX-18MV stopped-flow apparatus equipped with a diode array detector from Applied Photophysics. The optical quartz cell with a path length of 10 mm had a volume of 20 μL. The fastest time for mixing was 1 ms, and all measurements were performed at 25 °C. The ferric protein (2 μM) in 50 mM phosphate buffer (pH 7.0) was mixed with either 2 or 25 μM H_2_O_2_ and measured in triplicate for 10 and 100 ms. Second-order rate constants (*k*_app_) for the formation of Compound I were calculated using ProK IV global fitting software assuming a pseudo-first-order reaction and the model A + B > C.

### Molecular Dynamics Simulations and Analysis

Molecular dynamics simulations of wild-type *Kp*DyP were performed using the GROMOS11 molecular simulation package^36^ and GROMOS force field 54A7^37^ and based on crystal structures with PDB entries 6FKS (*Kp*DyP), 3O72 (EfeB), 4W7J (*Aau*DyP), 3Q3U (lignin peroxidase), and 1ZBY (C*c*P). The heme *b* cofactor was linked to the coordinating histidine H215,^38^ and the structure was first relaxed by an in vacuo steepest descent energy minimization with a convergence criterion of 0.1 kJ/mol. These energy-minimized structures were used for initial calculations of the decay factor using the GROMOS++ package *epath*.^39^ The program *epath* uses Dijkstra’s graph search algorithm to find the electron pathway with the highest product of the decay factors, corresponding to the “shortest path”.^40^ The decay factor *q*_*ij*_ for the electron transfer between atoms *i* and *j* is calculated according to
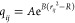
where *r_ij_* is the distance between the atoms and the parameters *A*, *B*, and *R* are specified for jumps through covalent bonds, hydrogen bonds, and space as described by Beratan et al.^41^ MD simulations of *Kp*DyP have been described previously.^28^ In short, the protein was solvated in a periodic rectangular simulation box with the simple point charge water model^42^ (minimal solute–wall distance of 0.8 nm), subjected to a second energy minimization step to remove unfavorable solute–solvent contacts. To obtain a neutral system and mimic, a 50 mM buffer (pH 7) with 30 sodium and 21 chloride ions was added to the system prior to equilibration (gradual temperature increases of 60 K every 20 ps followed by 100 ps at 300 K and constant pressure).

Plain MD simulations (300 K and 1 atm) were performed for 30 ns, using a step size of 2 fs. Coordinates were written out every 0.5 ps. This was achieved through weak coupling with a relaxation time of 0.1 ps for the temperature and 0.5 ps for the pressure.^43^ The isothermal compressibility was set to 4.575 × 10^–4^ (kJ mol nm^–3^)^−1^. Bond lengths were constrained to their optimal values with a relative geometric accuracy of 10^–4^ using the SHAKE algorithm.^44^ The nonbonded interactions were calculated using a twin-range cutoff^45^ and a molecular pair list, with a short-range cutoff of 8 nm and a long-range cutoff of 1.4 nm. A reaction-field contribution^46^ was added to the electrostatic interactions and forces to account for a homogeneous medium outside the cutoff using a dielectric permittivity of 61.^47^ Analyses of the coordinate trajectories were performed with Gromos++ programs *hbond*, *rdf*, *epath*, and *mdf*.^39^

### Electron Paramagnetic Resonance (EPR) Spectroscopy

All samples for low-temperature EPR spectroscopy (enzyme concentration of ≈500 μM) were prepared in 50 mM phosphate buffer with 25% glycerol as a cryoprotectant. The protein is being activated by addition of a 5-fold molar excess of H_2_O_2_, and the incubation time between manual admixing and flash freezing in liquid N_2_ is approximately 30 s. Continuous-wave (CW) EPR at X-band (∼9.44 GHz) is performed on a Bruker ESP300E instrument (Bruker Biospin) equipped with a liquid helium cryostat (Oxford Instruments) enabling temperatures from 2.5 K to room temperature. Calibration of the magnetic field was done using a Bruker ER035 M NMR Gaussmeter. Broad-field spectra at 10 K were recorded using a microwave power of 1 mW and an amplitude modulation of 0.5 mT. Narrow-field spectra of the radical were recorded at 80 K using a microwave power of 0.1 mW and a modulation amplitude of 0.1 mT. A modulation frequency of 100 kHz was used, and all samples were vacuum-pumped to 1 mbar to remove excess paramagnetic dioxygen.

An attempt was made to quench the resting-state radical using ascorbic acid and ABTS [2,2′-azino-bis(3-ethylbenzothiazoline-6-sulfonic acid)]. The substrate was added to the ferric protein in a 40-fold stoichiometric excess. After being incubated for a few minutes at room temperature, the samples were transferred in quartz tubes and flash frozen in liquid N_2_.

Pulsed EPR experiments at X-band (9.74 GHz) were performed on a Bruker E580 ElexSys spectrometer (Bruker Biospin) equipped with a gas-flow cryogenic system (Oxford Instruments). Hyperfine sublevel spectroscopy (HYSCORE) is performed at *g* ≈ 2.005 using the π/2−τ–π/2–*t*_1_–π–*t*_2_–π/2−τ–echo sequence with π/2 (π) pulse lengths of 12 (24) ns and *t*_1_ and *t*_2_ ranging from 96 to 4896 ns in steps of 16 ns.^48^ HYSCORE spectra were recorded using a four-step phase cycle, and postprocessing includes baseline correction of the real part of the time traces with a third-order polynomial. The result is apodized with a Hamming window and zero-filled before Fourier transformation and calculation of the absolute value spectrum. HYSCOREs using two τ values (104 and 184 ns) are averaged according to their respective noise levels.

Q-Band (∼ 34 GHz) CW EPR spectroscopy was performed on a Bruker ElexSys E500 spectrometer (Bruker Biospin), equipped with a CF910 He-flow (Oxford Instruments) cryostat (2–4300 K). A Pendulum CNT-90XL frequency counter and a Bruker ER035M NMR Gaussmeter were used to measure the microwave frequency and magnetic field, respectively. Spectra of the resting-state radical at 80 K were recorded using a microwave power of 0.1 mW, a modulation amplitude of 0.5 mT, and a modulation frequency of 10 kHz. At W-band (∼ 94 GHz), we performed electron spin echo (ESE)-detected EPR spectroscopy on a Bruker ElexSys E680 spectrometer (Bruker Biospin) equipped with a standard single-mode cylindrical resonator from Bruker and a continuous-flow cryostat and superconducting magnet (Oxford Instruments). A π/2−τ–π–τ–echo sequence is used for the ESE-EPR at 20, 40, 60, and 80 K using π/2 (π) pulse lengths of 120 (240) ns. The experiment is averaged over two τ values (340 and 400 ns), and a shot repetition time of 5000 μs is used. The W-band magnetic field was calibrated using a Mn(II)-doped MgO powder sample. CW EPR at 275.7 GHz was obtained on a spectrometer developed at the Huygens-Kamerlingh Onnes Laboratory (Leiden University, Leiden, The Netherlands)^49^ with a probe dedicated to operation in CW mode.^50^ The resting-state radical spectrum was recorded at 80 K using a microwave power in the microwatt range, a modulation amplitude of 0.4 mT, and a modulation frequency of 1.7 kHz.

### Electronic Circular Dichroism (ECD) Spectroscopy

To assess the structural changes of the created single mutants and double mutants, electronic circular dichroism (ECD) spectra in the far near (260–310 nm) UV regions were recorded (Chirascan, Applied Photophysics, Leatherhead, U.K.). The following settings were used: spectral bandwidth of 1 nm, scan speed of 10 s nm^–1^, path length of 1 cm, and temperature of 20 °C. Samples were prepared with a final concentration of 10 μM in 5 mM phosphate buffer (pH 7).

## Results

### *Kp*DyP Contains a “Resting-State” Radical Site Composed of Two Distinct Species

**Figure 2:**
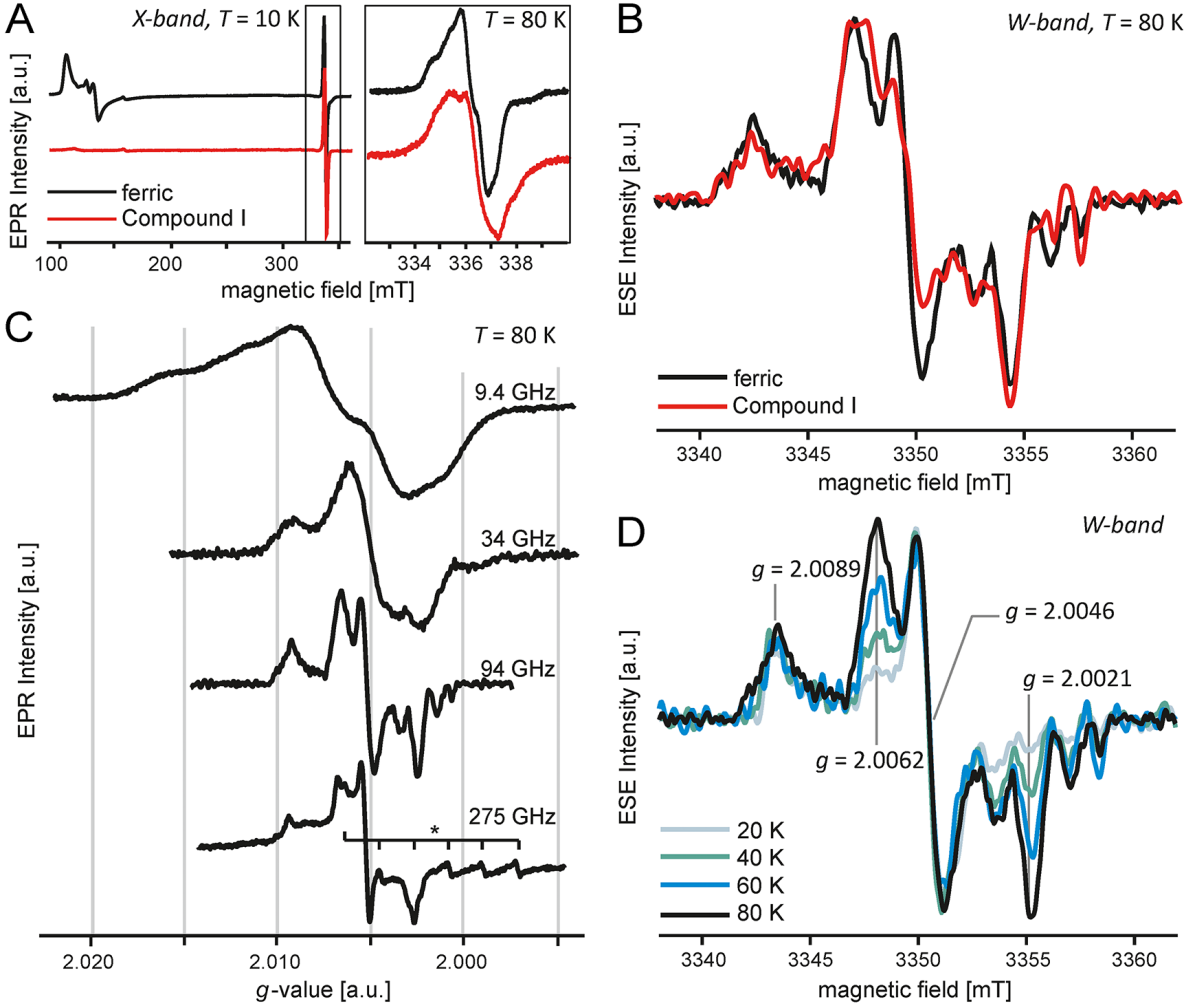
Multifrequency EPR of frozen protein solutions of wild-type *Kp*DyP showing the resting-state radical species consisting of two distinct radicals. (A) X-Band CW EPR spectra of wild-type *Kp*DyP before (black) and after (red) addition of H_2_O_2_ to form Compound I at 10 K and (box) a close-up of the radical species at 80 K. (B) First derivative of W-band ESE-detected EPR (τ = 340 and 400 ns) of the radical species at 80 K. The spectra are shown normalized for comparison. (C) CW EPR at Q-band (34 GHz) and J-band (275 GHz) and first derivative of W-band ESE-detected EPR (94 GHz; τ = 340 and 400 ns) of the radical species in wild-type *Kp*DyP at 80 K. Gray lines correspond to the conserved positions at the *g* values indicated in spectrum (D). The asterisk indicates a cavity background signal from Mn(II). (D) First derivative of W-band ESE-detected EPR (τ = 340 and 400 ns) of the radical species at 20, 40, 60, and 80 K.

The EPR spectrum of ferric wild-type *Kp*DyP indicates the presence of an organic radical signal (Figure 2A). While we have observed a similar radical feature in *Ec*DyP (YfeX), the homologue from *E. coli* (Figure S1), this has not been reported for other heme peroxidases so far, including B-type DyPs.^32^ Importantly, the radical was not observed in the catalytically inactive alanine variants D143A and R232A of *Kp*DyP, suggesting its relation with the peroxidase cycle.^11^

The tyrosine and tryptophan residues are predominantly localized in a Y-W belt along the interface of the central β sheets (Figure 1E). Five tyrosine residues (Y82, Y92, Y251, Y256, and Y237) appear to be solvent accessible and thus potential candidates for surface oxidation sites. Tyrosine 237 represents the structurally conserved tyrosine residue described above that acts as an electron entry site in other DyPs (Figure 1C).^8,24,34,35^ Furthermore, W176 may be a candidate for spontaneous electron transfer due to its proximity to the heme *b* periphery (Figure 1A).

First, we have analyzed the EPR spectral features of wild-type *Kp*DyP with and without H_2_O_2_ activation. Using CW EPR spectroscopy at 2.5 K and a saturating microwave power, a stable Compound I porphyrin π-cation radical was observed upon reaction with a 5-fold excess of H_2_O_2_.^11^ Unexpectedly, this did not induce a major change in the observed radical species, as one can see in X-band CW EPR measurements at 80 K, a temperature at which the porphyrin π-cation radical can no longer be observed (Figure 2A). The inset of Figure 2A indicates an identical position of the central line but reveals a changing substructure. At W-band (94 GHz), electron spin echo (ESE)-detected EPR spectroscopy was performed using two different interpulse times (different τ values), averaged, and smoothed before taking the first derivative yielding a CW-like EPR spectrum (Figure 2B). Here, the improved spectral resolution shows small changes in the resting-state radical upon H_2_O_2_ activation, but all features remain conserved at identical *g* values.

To determine the location of the resting-state radical site, we performed a series of low-temperature EPR experiments, which can be used to distinguish protein radicals.^51−53^ Tyrosine and tryptophan residues can usually be distinguished by their **g** tensor, as shown for *E. coli* ribonucleotide reductase and photosystem II.^54−57^ While both exhibit a *g*_*z*_ value close to the free electron *g*_e_, the *g*_*x*_ and *g_y_* values are governed by spin–orbit coupling (with increasing spin density on heavier atoms) giving rise to a smaller anisotropy for tryptophan radicals (*g*_*x*_ ≈ 2.0035) than for tyrosine radicals (*g*_*x*_ values typically between 2.006 and 2.0092).^52^ Unfortunately, the obtained X-band CW EPR spectra of the radical were strongly obscured by line broadening (Figure 2A). To circumvent the hyperfine broadening of spectral lines, we turned to multifrequency EPR to increase the resolution and identify the *g* principal values. EPR spectroscopy was repeated at Q-band (34 GHz), W-band (94 GHz), and J-band (275 GHz). ESE-detected EPR spectra are displayed as a first derivative for ease of comparison (Figure 2C).

The conserved signal at ∼ 2.0089 in the Q- to J-band spectra indicated the presence of at least one tyrosyl radical (Figure 2D). Moreover, a comparison of the first derivative of the W-band ESE-detected EPR spectra recorded at different temperatures indicates that the resting-state radical is composed of two distinct radical sites with different temperature-dependent relaxation times (Figure 2D). The signals at *g* values of 2.0062 and 2.0021 seem to be correlated. A maximal *g* value (*g*_max_) of 2.0089, in combination with the central *g* value of 2.0046, suggests the presence of a tyrosyl radical with no hydrogen bonding (Figure 2D).^52^ The origin of the *g* feature at 2.0062 defies a clear-cut interpretation.

EPR spectra of tyrosyl and tryptophanyl radicals are strongly governed by the local protein environment determining the *g* values.^58^ Although hydrogen bonding to the C=O group of tyrosine residues generally involves decreasing *g*_*x*_ to ∼2.0076,^52^ which is close to the *g*_*x*_ of 2.0075 found for Y337 in *Aau*DyP,^6^ it is significantly higher than 2.0062. Tyr radicals with similar *g* values have been found in yeast C*c*P (*g*_max_ = 2.0066),^14,59^ KatG from *Synechocystis* PCC6803 (*g*_max_ = 2.0064),^60^ and turnip isoperoxidase 7.^61^ In γ-irradiated l-tyrosine HCl crystals, a Tyr radical with strong hydrogen bonding has been identified with a *g*_max_ of 2.0062.^62^ Tryptophanyl radicals, on the contrary, display much lower *g*_max_ values and, even at W-band, a hardly distinguishable **g** tensor. If their spectrum is dominated by proton hyperfine couplings, as is the case for W337^•^ in *Aau*DyP, identification by spectral simulation is possible.^25^ Although again rather unusual, a feature around *g* = 2.0062 can occur for a tryptophanyl radical with a major proton hyperfine coupling. Indeed, while the protons of the aromatic ring exhibit fixed hyperfine interactions, a broad range of values is observed for the β protons depending on the side-chain orientation.^63,64^ This explanation, however, is contradicted by the observation that the peak remains at the same *g* value (2.0062) in the W-band and J-band EPR spectra and also falls within the broad central feature of the Q-band EPR spectrum. This indicates that the peak corresponds to a principal *g* value and is not hyperfine-related.

Unfortunately, pulsed EPR methods, such as electron–nuclear double resonance (ENDOR),^65−67^ could not provide additional information as the high-spin Fe(III) signal from heme *b* strongly hampers the application of hyperfine techniques below 20 K. As the ESE intensity of the high-spin Fe(III) drops to zero at higher temperatures, we collected X-band hyperfine sublevel correlation spectroscopy (HYSCORE) data at 40 K and a magnetic field corresponding to a *g* value of ≈ 2.005. Cross peaks centered around 2.5 MHz indicate the presence of a weak nitrogen hyperfine coupling (Figure S2), which can be equally well explained by the presence of either a tryptophanyl radical or the interaction of a tyrosyl radical in which the unpaired electron is hyperfine coupled to the ^14^N nucleus from the protein backbone. At 14.7 MHz, a ridge due to ^1^H couplings can be clearly distinguished. Unfortunately, unlike the HYSCORE spectra of the tyrosyl radical in photosystem II and bovine liver catalase, these features were not sufficient for a complete assignment of the proton hyperfine tensors.^68^

### Rational Selection of Tyrosine and Tryptophan Residues for Site-Directed Mutagenesis

**Figure 3:**
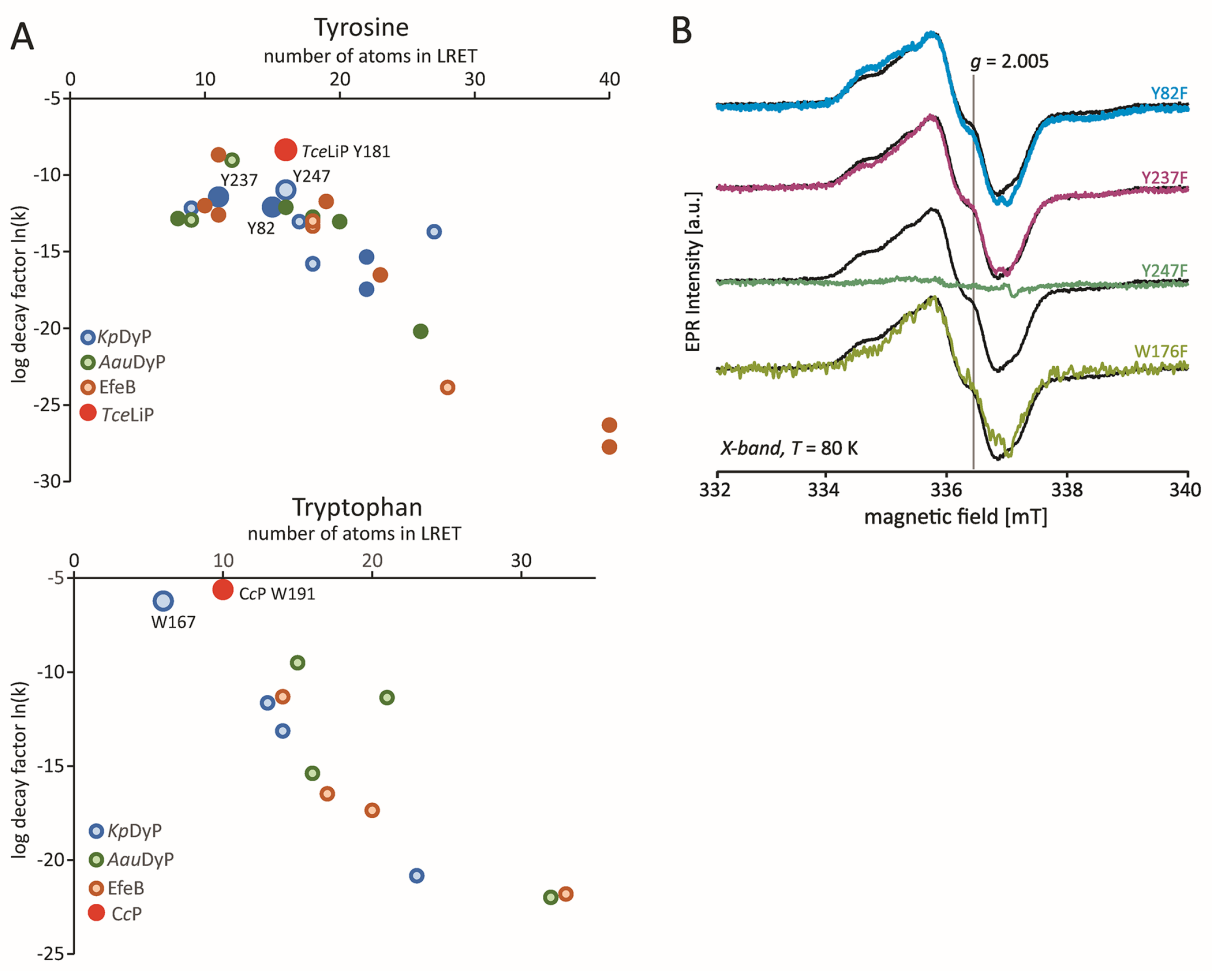
Y247 harbors the dominant resting-state radical site. (A) *epath* calculation for all Tyr and Trp residues of *Kp*DyP (blue), *Aau*DyP (green), EfeB (orange), and C*c*P W191/*T. cervina* lignin peroxidase (*Tce*LiP) W181 (red). Residues with surface exposure (determined from crystal structure) are shown as filled circles. (B) X-Band CW EPR spectra at 80 K of the radical species in *Kp*DyP variants Y82F (light blue), Y237F (magenta), Y247F (green), and W176F (olive) in comparison with the wild-type protein (black).

As EPR spectroscopy of the resting-state radical in wild-type *Kp*DyP alone did not allow clear identification of the involved amino acids, we continued with computational analysis of LRET paths followed by the design and recombinant production of variants with exchanged tyrosine and tryptophan residues (Figure 1). First, on the basis of the crystal structure, we calculated potential electron transfer paths using the GROMOS++ program *epath* to score all tyrosine and tryptophan residues in *Kp*DyP (PDB entry 6FKS) (Figure 3A).^39,69^ Additionally, we calculated the decay factor for all residues in A-type EfeB from *E. coli* (PDB entry 3O72)^70^ and D-type *Aau*DyP (PDB entry 4W7J).^6,7^ The well-characterized catalytically essential radical sites of lignin peroxidase from *T. cervina* (Y181, PDB entry 3Q3U)^4^ and C*c*P from *Saccharomyces cerevisiae* (W191, PDB entry 1ZBY)^12^ were used for comparison. The program *epath* calculates the minimal decay factor for the transfer of an electron from the designated donor atom to an acceptor atom based on the parameters for electron jumps through covalent bonds, hydrogen bonds, and space defined by Beratan et al.^41^ In principal, the electron will be donated at the closest edge of the porphyrin. Here, for the sake of simplicity, we calculated the inverse route starting from the heme iron to the tyrosine OH atoms or central NE_1_ atom of tryptophans. As one can see in Figure 3A in all DyPs, the calculated decay factor, shown as ln(*k*), mostly correlates with the number of atoms involved as a measure of increasing distance. However, the catalytic radical sites in both lignin peroxidase and C*c*P have a much lower decay factor, i.e., ln(*k*) = −8.38 and −5.62, compared to those of the three DyP representatives (Table 1), although the numbers of atoms involved in the respective paths are in a similar range (13 atoms in lignin peroxidase and 10 atoms in C*c*P). The decay factors for the described radical site in *Aau*DyP W377 [ln(*k*) = −9.49], Y337 [ln(*k*) = −12.86], and Y147 [ln(*k*) = −12.13] and the likely radical site on EfeB Y352 [ln(*k*) = −11.75] were used as a cutoff for selection of target residues in *Kp*DyP.

**Table 1:** Logarithmic Decay Factors [ln(*k*)] Calculated Using *epath* and Numbers of Atoms Involved in the Potential Electron Pathways of *Kp*DyP, EfeB, *Aau*DyP, *Tce*LiP, and C*c*P^a^

tyrosine			tryptophan		
residue	no. of atoms	decay factor ln(*k*)	residue	no. of atoms	decay factor ln(*k*)
*Kp*DyP					
**247**	**16**	–10.98 (−11.93)	**176**	**6**	**–6.23**
**237**	**11**	–11.45 (−12.49)	132	13	–11.64
**82**	**15**	–12.10 (−12.73)	18	14	–13.13
287	9	–12.18	63	23	–20.83
251	17	–13.07			
256	27	–13.73			
170	22	–15.37			
288	18	–15.81			
92	22	–17.47 (−17.38)			
EfeB					
*273*	*11*	*–8.69*	240	14	–11.31
**352**	**19**	**–11.75**	166	17	–16.47
360	10	–12.01	211	20	–17.36
310	11	–12.61	221	33	–21.81
329	18	–13.03			
308	18	–13.36			
227	23	–16.52			
84	28	–23.87			
15	40	–26.32			
373	40	–27.76			
*Aau*DyP					
*285*	*12*	*–9.05*	**377**	**15**	**–9.49**
**147**	**16**	**–12.13**	256	21	–11.35
362	18	–12.77	105	16	–15.38
**337**	**8**	**–12.86**	207	32	–21.99
433	9	–12.95			
229	20	–13.06			
25	26	–20.21			
*Tce*LiP, tyrosine			C*c*P, tryptophan		
**181**	**16**	**–8.38**	**191**	**10**	**–5.62**

a Selected residues in *Kp*DyP and described/predicted radical sites are highlighted in bold, and LRET paths using the artificial His–iron link (italics) were not considered. The decay factors calculated from 30 ns MD simulations are given in parentheses.

On the basis of the calculations presented above, we selected three tyrosine residues (Y82, Y237, and Y247) and one tryptophan residue (W176) for exchange with phenylalanine. Tyrosine 82 and Y237 are surface-exposed and thus potential oxidation sites. Tyrosine 237 is located at the previously described site conserved in A- and D-type DyPs (Figure 1C). Tryptophan 176 is located at the heme periphery (Figure 1A, W176–porphyrin distance of ∼ 3.5 Å). It may be partially exposed to the solvent sphere in the active site and has a decay factor comparable to that of C*c*P [ln(*k*) = −5.62]. Tyrosine 247 was selected as it has the lowest overall decay factor [ln(*k*) = −10.98] of all tyrosine residues. It is located in the proximity of the surface-exposed Y237 but is, according to the crystal structure, completely solvent inaccessible.

The selected variants Y82F, Y237F, Y247F, and W176F were recombinantly produced and biochemically analyzed for proper folding and heme insertion as described recently.^9^ Further variants produced during this project are W18F, Y92F, and the double mutants W18F/Y92F and Y92F/Y247F (see below). All variants exhibited UV–vis and EPR spectral characteristics of the iron(III) high-spin resting state similar to wild-type *Kp*DyP (Figure S3).

The organic radical signature remained in variants Y82F, Y237F, and W176F. Here, X-band CW EPR spectra (Figure 3B) show small changes that can be attributed to differences in the relative abundancy of the two radical species [confirmed by the first derivative of W-band ESE-detected EPR spectra (Figure S4)]. However, exchange of Y247 resulted in a major loss of radical intensity (Figure 3B), suggesting that theY247 radical mainly contributes to the EPR signal of the resting state of *Kp*DyP.

### A Tyr-Trp Dyad Stabilizes the Resting-State Organic Radical and Contributes to Catalytic Efficiency

The major decrease in the EPR intensity of the radical in the variant Y247F may be attributed to the loss of the main contribution of the composite resting-state radical (Figure 2D). We therefore assigned this contribution to Y247. As described above, we observed two features, one related to a *g*_max_ value of 2.0089 and one with a *g*_max_ of 2.0062 (Figure 2D). For tyrosyl radicals, a well-established relation has been found correlating *g*_max_ with the local electrostatic environment of the C=O group.^55,56,71,72^ Hence, Y247**^•^** could either be in a hydrophobic pocket (*g*_max_ = 2.0089) or exhibit hydrogen bonding with another residue (*g*_max_ = 2.0062).^73^ Unfortunately, the low intensity of the residual radical species in the Y247F variant (Figure 3B) did not allow in-depth analysis of the remaining signal using high-field EPR.

**Figure 4:**
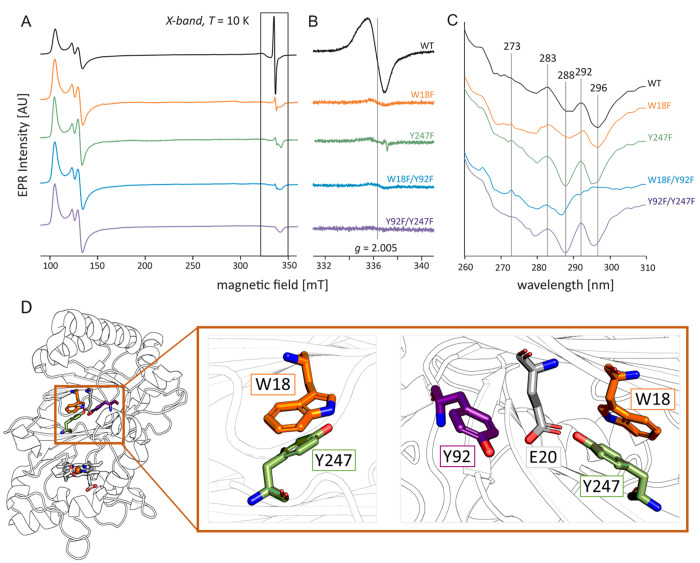
Tyrosine-tryptophan dyad that is required to stabilize the resting-state radical site at Y247. (A) X-Band CW EPR spectra at 10 K and (B) a close-up of the radical species at 10 K in resting-state WT *Kp*DyP (black) and the four variants: W18F (orange), Y247F (green), W18F/Y92F (blue), and Y92F/Y247F (violet). (C) Near-UV electronic circular dichroism spectra of the wild type (black), W18F (orange), Y247F (green), W18F/Y92F (blue), and Y92F/Y247F (violet). (C) X-Band CW EPR spectra at 80 K and (D) localization of Y247, W18, E20, and Y92 in one *Kp*DyP subunit (chain A). The inset shows close-ups of the Y-W dyad (left) and in context with Y92 and E20 (right). All residues and the heme *b* cofactor are shown as sticks, and the protein backbone is shown as a cartoon outline.

In the crystal structure of *Kp*DyP, the indole ring of W18 is located in a displaced parallel orientation to Y247 (Figure 4D). The distance between the center of the phenyl ring of Y247 and the indole ring of W18 is 4.15 Å, within range for π–π stacking interaction and/or charge resonance stabilization. Thus, we further investigated the effect of an exchange of W18 with phenylalanine on the radical signature of Y247. The X-band CW EPR radical signature of variant W18F is similar to that obtained for Y247F (Figure 4A,B), indicating that W18 is involved in stabilization of the radical site in Y247. A remaining weak contribution was again observed, showing that W18 itself does not harbor a radical site. Interestingly, the LRET path calculated for Y92 included Y247. Although Y92 is an unlikely candidate as it is located far from heme *b*, it has the lowest calculated decay factor [ln(*k*) = −17.47] and it is not conserved among the different DyPs. Surprisingly, the Y92F/Y247F double variant did not contain any detectable residual radical signature, thus identifying Y92 as the second radical site (Figure 4A,B). The radical in the W18F/Y92F variant is attributed to the presence of Y247. It is much weaker than in the wild-type protein, because the stabilization with W18 is missing.

As described above, EPR indicates the presence of at least one hydrogen-bonded tyrosyl radical. Figure 4D shows the structure of the Y247-W18 dyad and Y92, which are bridged by E20. Glutamate 20 is within hydrogen bonding distance of Y247-OH and, depending on the overall flexibility in this region, Y92. To further investigate the H-bond occurrence and dynamics, we performed molecular dynamics simulations of wild-type *Kp*DyP for 30 ns. The residues in H-bonding proximity (here assumed as a distance of < 0.25 nm, an angle of > 135° between the hydrogen of the donor atom and the acceptor atom) of the OH atoms of Y247, Y92, and Y237 are listed in Table 2. For 90% of the simulation time, the OE2 atom of E20 is within H-bonding distance of Y247. In contrast, the E20 OE1 atom is within hydrogen bonding distance of Y92 for only 10% of the simulation time. In addition, Y237 is predominantly hydrogen bonding to the backbone oxygen of W176 (60%).

**Table 2:** Hydrogen Bonds of Y247, Y92, and Y237 in *Kp*DyP^a^

residue	atom	distance	angle	no. of occurrences	% occurrence
Y247					
Glu20	OE2	0.173	167.6	19708	98.54
Glu20	CD	0.236	147.4	9801	49
Glu20	OE1	0.184	164.4	263	1.31
Y92					
Glu20	OE1	0.174	165.3	2051	10.26
Y237					
Trp176	O	0.175	164.9	11952	59.76
Glu177	OE2	0.181	165.4	355	1.77
Trp176	C	0.244	154.6	247	1.23

a Interatomic distances, angles, and frequencies of occurrence of the Y-OH group to acceptor atoms during a 30 ns plain MD simulation of wild-type *Kp*DyP.

Next, to analyze the alteration in the local structure of Y247, Y92, and W18 caused by the mutations, we performed ECD spectroscopy of the fingerprint area (near-UV, 250–310 nm), which reflects spectral features of aromatic residues (Figure 4C). The near-UV spectra of the different variants may reflect both the exchange of the respected amino acid and changes in the interaction with neighboring (aromatic) residues (i.e., H-bonding, solvent exposure, and polarizability).^74^ While phenylalanine contributes only in the lower-wavelength range up to 260 nm, the signal from tyrosine and tryptophan residues may overlap significantly. Indeed, chiral tryptophan model peptides have been reported to have a stronger negative ellipticity in the region from 260 to 290 nm than the same peptides with tyrosine, with the negative ellipticity signal from tryptophan extending to 305 nm, while tyrosine does not contribute in this region.^75^ In variants W18F and W18F/Y92F, the overall signal intensity is decreased. Spectral similarities between W18F and the wild-type protein suggest that W18 itself does not contribute significantly to the overall ellipticity in wild-type *Kp*DyP, indicating an achiral environment that is not disturbed by the mutation. In variants Y247F and Y92F/Y247F, however, the ECD spectra change significantly compared to that of the wild-type protein. In Y247F and Y92F/Y247F, the overall negative ellipticity is increased with dominant features at 292 nm (positive) as well as 288 and 294–296 nm (negative). The signal at 294–296 nm is lost in W18F/Y92F and exhibits a reduced intensity in W18F. Assuming that F18 can interact in a π–π stacking mode with Y247 similar to W18 and the hydrogen bond between E20 and Y247 is present, the overall structure in W18F and W18F/Y92F remains undisturbed. The loss of signal intensity of these variants can thus be attributed to the exchange itself, as phenylalanine does not contribute at this wavelength.^74^ In both Y247F and Y92F/Y247F, the hydrogen bonding network described above is lost, which may increase the chirality of the neighboring W18 and may account for the higher ellipticity in these variants. Additional changes can be observed at 273 nm (peak in W18F, Y247F, and W18F/Y92F) and 283 nm (loss of signal intensity in W18F and W18F/Y92F) with an additional shoulder at 281 nm in W18F and W18F/Y92F.

**Figure 5:**
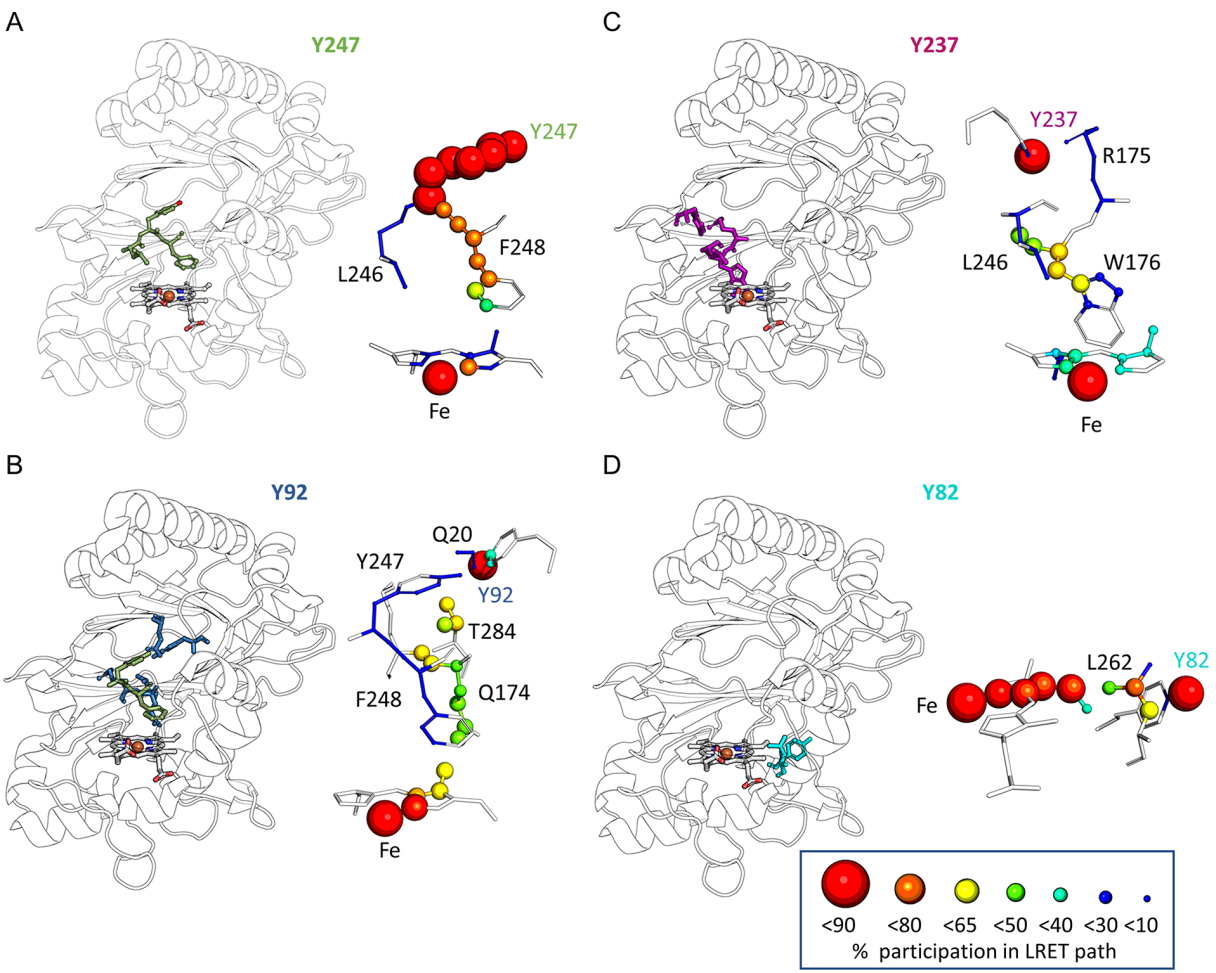
Location and structure of the calculated LRET paths to (A) Y247, (B) Y237, (C) Y92, and (D) Y82. The heme *b* cofactor and the residues involved in the respective paths are shown in a stick-and-ball representation (left), and the overall structure is shown as a cartoon (black outline). All atoms found in the LRET paths are shown on the right, with the frequency of their participation highlighted by sphere size and color code (the detailed code used is shown in the inset at the bottom left corner), and connecting sticks are colored according to sphere color.

**Figure 6:**
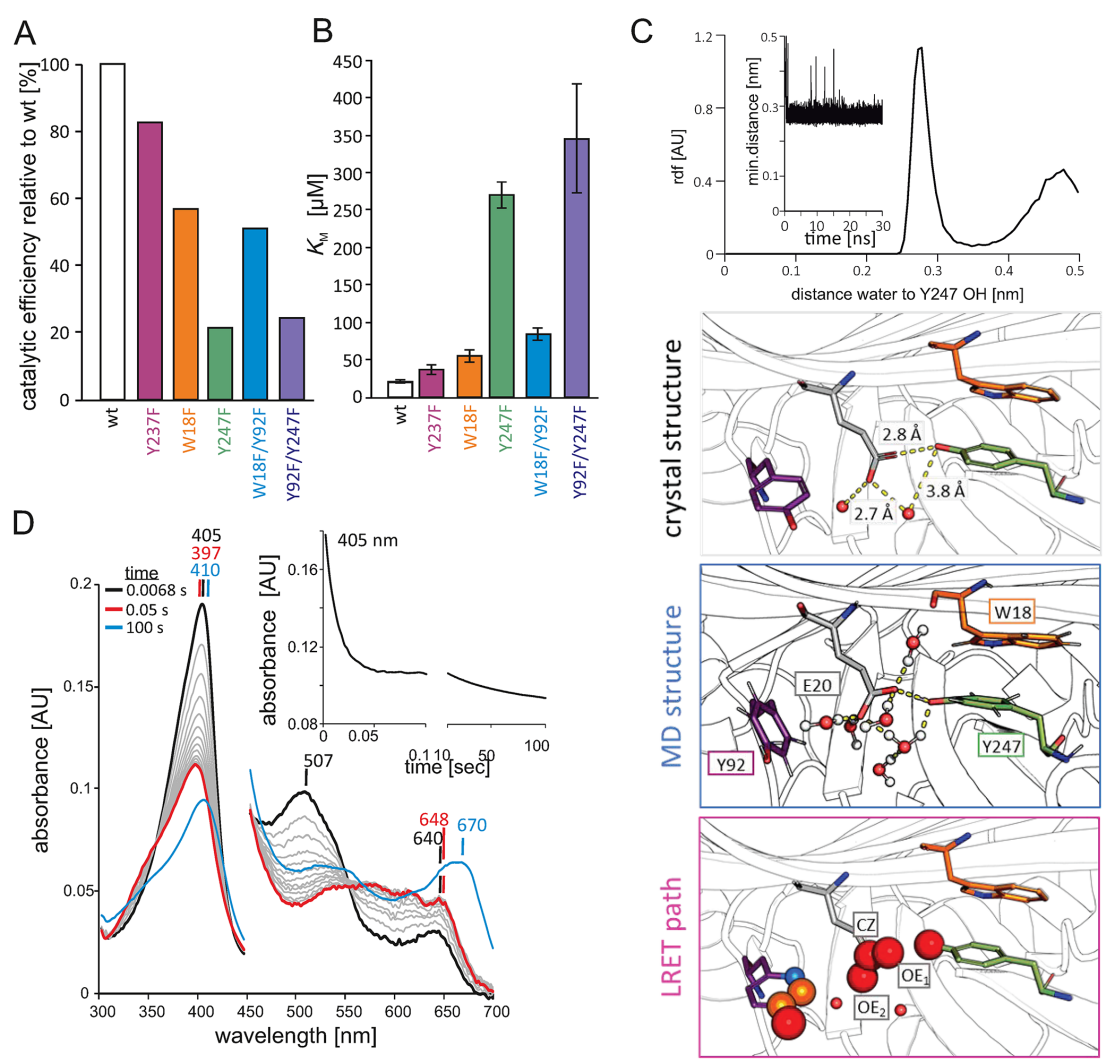
Tyrosine variants show different activity toward ABTS. (A) Catalytic efficiency (*k*_cat_/*K*_M_) of all variants as a percent relative to wild-type *Kp*DyP. (B) *K*_M_ values of the wild-type protein and variants toward ABTS. (C) Radial distance distribution of water relative to Y247 OH (top). The inset shows the minimum distance of water to Y247 OH over 30 ns. Boxes are structural representations of Y247, W18, E20, and Y92 and the closest waters in the crystal structure (gray box), the MD simulation (representative structure in the blue box), and the electron path of Y247, E20, and Y92 (pink box) with radii and colors as in Figure 5. (D) Compound I formation and decay of 2 μM Y247F with 25 μM H_2_O_2_. The resting state is colored black, Compound I red, and the final spectrum after 100 s blue. The inset shows the time trace at 405 nm.

The frequency and stability of electron transfer along specific pathways are sensitive to small changes in interatomic distances if electron jumps through space and along hydrogen bonds contribute. We therefore probed whether the initial scoring performed with the rigid structurally condensed proteins at their respective energy minima is an adequate estimate of the LRET stability and likelihood. Figure 5 shows the location of the LRET paths calculated for Y247, Y92, Y237, and Y82 as well as the involved residues scaled and colored with respect to the frequency of their participation in the LRET paths (> 90%, red spheres; 80–90%, orange spheres; 65–80%, yellow-green spheres; 50–65%, turquoise; 40–50%, cyan; 30–40%, dark blue spheres; 10–30%, dark blue no spheres; < 10%, white). Small fluctuations in interatomic distances throughout the simulations may lead to alterations in the electron transfer routes. Hence, all paths contain more contributing atoms compared to the calculation performed with the rigid structures. The calculated decay factors were lower for all residues, but the Y247 > Y237 > Y82 > Y92 hierarchy was identical and correlated with the number of atoms involved. The LRET path for Y247 is the most likely [ln(*k*) = −11.93; 53 atoms] with only one jump through space and the most likely route along covalent bonds of F248 and Y247 (Figure 5A). The path to Y237 scored again slightly lower [ln(*k*) = −12.49; 92 atoms], requiring two jumps through space: between heme *b* and the W176 indole ring and W176 backbone oxygen and Y237-OH (Figure 5B). Electron transfer from Y82 would include two space jumps: between Y82 and L262 and heme *b* [ln(*k*) = −12.73; 120 atoms] (Figure 5D). Direct electron transfer from Y92 has a low probability [ln(*k*) = −17.368; 196 atoms]. The route identified most often does not use Y247 but runs along Q174 with one space jump from T284. A route including Y247 by consecutive jumps along H-bonds among Y247, E20, and Y92 was found with a frequency of 20–25% (Figure 5B). Unsurprisingly, a direct electron transfer between Y92 and Y247 would always use the OE1-CZ-OE2 side chain of E20 [decay factor ln(*k*) = −6.59; 19 atoms] (Figure 6C, pink box).

### Tyr-247 Is Involved in Substrate Oxidation

Oxidation of the peroxidase model substrate 2,2′-azino-bis(3-ethylbenzothiazoline-6-sulfonic acid) (ABTS) by *Kp*DyP was previously reported (*k*_cat_/*K*_M_ = 1.5 × 10^5^ M^–1^ s^–1^; *K*_M_ = 20.4 ± 1.9 μM).^11^ The crystal structure of *Kp*DyP reveals two possible access routes to the heme *b* cofactor. One, suggested as the access route for H_2_O_2_, is narrow (bottleneck radius of < 3 Å) and is unlikely to accommodate larger substrates such as ABTS. The second access is via the solvent-exposed propionate p7. As one can see in Figure 1E, Y92 is solvent-exposed while Y247 appears to be buried but is in the proximity of the protein surface (Figure 4D). As was observed for other DyPs, ABTS is more efficiently oxidized at acidic pH values. We therefore measured ABTS oxidation at pH 5, a pH at which the enzyme was shown to be stable.^11^ Previously, pH-dependent differences in radical formation were reported for *Aau*DyP. In *Kp*DyP, the radical signature in the resting state is identical at pH 5 and 7 (data not shown). Next, we analyzed the pre-steady-state kinetics of Compound I formation by stopped-flow UV–vis spectroscopy. Figure 6D shows the spectral transitions of Y247F upon addition of 25 μM H_2_O_2_, which are representative for all variants. None of the mutations influences Compound I formation. All variants showed second-order rate constants (*k*_app_) of Compound I formation (red spectrum) with H_2_O_2_ comparable to that of the wild-type protein [*k*_app_ ∼ 5–6 × 10^6^ M^–1^ s^–1^ (Table 3)]. The spectral conversion including ∼ 50% hypochromicity at the Soret region, a shift of the Soret maximum to 397 nm, and the appearance of a maximum at 648 nm mirrored the features of wild-type *Kp*DyP. No spectral evidence of a Compound I* was detected after 100 s. A new maximum at 670 nm indicates heme bleaching and concomitant degradation of the porphyrin ring as described previously (Figure 6D, blue spectrum).^11,76^

**Table 3:** Steady-State Kinetic Parameters of Wild-Type *Kp*DyP and Variants for Oxidation of ABTS at pH 5 and Second-Order Rate Constants for the Formation of Compound I (Cpd I) with H_2_O_2_

	*K*_M_ (μM)	*k*_cat_ (s^–1^)	*k*_cat_/*K*_M_ (M^–1^ s^–1^)	Compound I *k*_app_ (M^–1^ s^–1^)
WT	20.5 ± 1.9	3.1 ± 0.1	1.5 × 10^5^	(5.2 ± 0.2) × 10^6^
Y237F	36 ± 7	4.6 ± 0.4	1.25 × 10^5^	(5.7 ± 0.5) × 10^6^
W18F	54 ± 8	4.7 ± 0.4	8.6 × 10^4^	(5.2 ± 0.2) × 10^6^
Y247F	270 ± 16	8.7 ± 0.3	3.3 × 10^4^	(5.3 ± 0.3) × 10^6^
W18F/Y92F	84 ± 8	6.5 ± 0.4	7.7 × 10^4^	(5.9 ± 0.3) × 10^6^
Y247F/Y92F	345 ± 73	12.8 ± 1	3.7 × 10^4^	(5.1 ± 0.3) × 10^6^

With the exception of ABTS oxidation, wild-type *Kp*DyP, similar to other B-type DyPs, was previously shown to be a rather poor oxidant of conventional DyP substrates such as Reactive Blue 19 in a pH range where enzyme stability is guaranteed (pH > 4.5).^11,30,32^ We therefore used ABTS to assess the steady-state kinetics of the variants at pH 5. The *k*_cat_, *K*_M_, and *k*_cat_/*K*_M_ values are reported in Table 3. While Y237F and the variants containing the W18F mutation have a more wild-type-like *k*_cat_ (∼ 1.5–2-fold higher), Y247F and Y247F/Y92F exhibit 3–4-fold higher *k*_cat_ values compared to that of the wild-type protein. The effect on *K*_M_ was more pronounced as the Y247F and Y247F/Y92F variants have 13- and 17-fold higher *K*_M_ values compared to that of wild-type *Kp*DyP (Table 3 and Figure 6B), while the W18F variants were again more wild-type-like (≤ 4-fold higher). The catalytic efficiency *k*_cat_/*K*_M_ of Y247F and Y247F/Y92F is governed by higher *K*_M_ values and results in an 80% lower activity toward ABTS compared to that of wild-type *Kp*DyP (Figure 6A). Oxidation of Reactive Blue 19 was not influenced by exchange of Y247 (not shown).

From inspection of the crystal structure, Y247 does not appear to be solvent-exposed and accessible for substrate oxidation. The nearest water is at a distance of 3.8 Å (Figure 6C, gray box). We therefore calculated the minimum distance (mdf) and radical distance distribution (rdf) from the MD simulations for water around the Y247 OH group (Figure 6C, top). Indeed, throughout the simulation the closest water is at ∼ 2.7 Å (considering the oxygen), which is within H-bonding distance. Figure 6C shows a representative structure with water molecules surrounding Y247 and E20 and suggests that Y247 is more accessible in a dynamic system (i.e., in solution) (Figure 6C, blue box) than the crystal structure suggests (Figure 6C, gray box).

## Discussion

All available crystal structures of DyPs show highly restricted access routes to the active site, which contradicts accommodation of bulky substrates such as Reactive Blue 19, ABTS, or phenolic lignin model compounds.^6−8,24^ However, as DyPs can oxidize these substrates, the existence of surface oxidation sites, linked to the active site by LRET paths as in lignin peroxidases and versatile peroxidase, seems likely.^5,23,77^ Indeed, A- and C/D-type DyPs form Compound I*. Distinct radical sites have been identified in D-type *Aau*DyP and A-type *Sl*DyP, whereas B-type DyPs have repeatedly been reported to form a highly stable Compound I that does not convert to Compound I* and thus does not generate protein radicals.

Here we identified two separate radicals with distinct temperature-dependent relaxation and *g*_max_ at *g* values of 2.0089 and 2.0062 in ferric B-type *Kp*DyP. We assigned the *g*_max_ of 2.0089 to a non-hydrogen-bonded or weakly hydrogen-bonded tyrosyl radical. The decay factor ln(*k*) for electron transfer was calculated from static structures and used to select four candidate residues. These scored like the described radical sites in *Aau*DyP (Y147, Y337, and W377) and the likely radical site in EfeB (Tyr352, the same position as Y374 in *Sl*DyP and Y332 *Tc*DyP).

We correctly predicted Y247 as the main contributor to the bicomponent radical signature (Figure 2B). Although the crystal structure of *Kp*DyP indicates that Y247 is not solvent-exposed, it is located close to the surface and molecular dynamics simulations show that it can be accessed in a dynamic system (i.e., in solution) (Figure 6C, blue box). Its orientation and proximity to W18 would allow radical stabilization through π–π stacking or charge resonance stabilization. The latter, although it cannot be excluded, is disfavored by the absence of a detectable tryptophanyl radical species. The interaction between the radical site at Y247 and the W18 ^14^N nucleus may explain the weak ^14^N hyperfine coupling observed by HYSCORE spectroscopy (Figure S2). Indeed, we found this Y-W dyad to be required to maintain the stable resting-state radical. In MD simulations of the wild-type protein, Y247 is H-bonded to E20 for 90% of the time, linking it to the EPR radical signal with a *g*_max_ of 2.0062. Interestingly, the *g*_max_ of 2.0062 reported here is close to the lowest *g*_*x*_ ever observed in a tyrosyl radical, i.e., for Y356 in *E. coli* RNR (*g*_max_ = 2.00619).^78^ In *E. coli* RNR, density functional theory calculations attribute the low *g*_max_ to coordination of Y356 by a symmetrical cluster of two water molecules.

The origin of the second radical signature was found to be Y92. Y92F/Y247F is the only variant with no detectable radical species. Despite its low decay factor when considering the full path to the heme *b* cofactor, Y92 is connected to E20 and thus Y247 via a H-bonding network. MD simulations indicate the presence of a hydrogen bond to E20, albeit with a lower frequency (10% of the simulation time) compared to that of Y247. A direct transfer between Y92 and Y247 via E20 has a significantly higher likelihood [ln(*k*) = −6.59]. We therefore propose the tyrosyl radical to originate through this route.

Perturbation of the H-bonding network appears to have a strong impact on the local structure of the Y-W dyad as indicated by ECD spectroscopy. Tryptophan 18 is located in a mostly achiral environment as long as the hydrogen bond network among Y92, E20, and Y247 is undisturbed. In all other variants, the H-bonding network is disturbed. The increased ellipticity of the Y247F and Y92F/Y247F variants (where W18 is still present) indicates a more chiral environment. The striking loss of the signal at 298 nm in the W18F/Y92F variant on the contrary is likely due to the exchange (phenylalanine does not absorb in the region between 280 and 305 nm) as presumably the hydrogen bond between Y247 and E20 remains and F18 may be more rigid in the π–π stacking interaction.

The Y247 and Y92 “resting-state” radicals in ferric wild-type *Kp*DyP must derive from the peroxidase activity as they are not present in inactive alanine variants of the catalytic D-R pair, which is required for heterologous cleavage of H_2_O_2_ (i.e., enzyme activation) (*k*_1_ in Figure 1D).^11^ Unlike radicals observed in other DyPs, activation by H_2_O_2_ does not promote formation of the resting-state radical (Figure 2). No spectral characteristics of a Compound I* could be detected in wild-type *Kp*DyP. This also applies to all variants investigated in this study. Their kinetics of Compound I formation was wild-type-like, and activation by H_2_O_2_ did not increase the amount or induce a major change in the radicals already present in the resting state. The stability of Compound I was wild-type-like in all variants.

The π–π stacking of the Y247-W18 dyad might promote stabilization of the resting-state radicals but has no impact on Compound I stability. On the contrary, steady-state kinetic data indicate that ABTS oxidation is sensitive to perturbation in the H-bonding network around the Y247-W18 dyad. Removal of Y247 reduces the overall ABTS oxidation efficiency, as the observed 3-fold increase in *k*_cat_ values is offset by 10-fold increases in the respective *K*_M_ value. This suggests the presence of alternative substrate oxidation site(s). Wild-type *Kp*DyP exhibits a relatively high affinity for ABTS (*K*_M_ ∼ 20 μM) that is oxidized at Y247 with a turnover number of ∼ 3 s^–1^, which must be the rate-limiting step and could (partially) explain the high stability of the catalytically inactive Compound I. Upon elimination of Y247 (as in Y247F and Y92F/Y247F), the catalytic efficiency of ABTS oxidation is reduced but apparently possible at an alternative low-affinity (*K*_M_ ∼ 300 μM) site with a higher turnover number (*k*_cat_ ∼ 9 s^–1^). Similarly, A-type *Sl*DyP also has several electron entry sites, including a binding site for Reactive Blue 19 that is apart from that of ABTS.^24^ Incidentally, A-type *Sl*DyP also lacks the *Aau*DyP W377 radical identified as the main catalytic site for efficient degradation of Reactive Blue 19 degradation.^7^ The location of the second oxidation site in *Kp*DyP remains to be determined.

In summary, we have identified an LRET path from heme *b* to two radical sites, which are connected by a H-bonding network and a stabilizing Tyr-Trp dyad. This dyad is present in only B-type DyP homologues from *E. coli* and *Enterobacter lignolyticus.* The absence of a similar radical in the EPR spectrum of B-type DyP from *R. jostii* may be explained by the presence of a phenylalanine instead of the stabilizing W18. In two other structures available to date (from *Vibrio cholerae* and *Shewanella oneidensis*), a tyrosine occupies the position of W18 while instead of Y247 a threonine or cysteine is present, respectively. Although we can only speculate about the origin of these radicals, we suggest that they are remnants of the enzymatic turnover of this enzyme. It is reasonable to assume that a low percentage of catalytic radical sites at protein surfaces is left over in all peroxidases that use LRET paths due to imbalance in H_2_O_2_ and electron donor availability. However, in general, these surface-exposed radicals are reactive and readily quenched unspecifically (and thus not observable), whereas in *Kp*DyP, the described dyad apparently stabilizes the resting-state radicals and thus renders it unreactive as evidenced by our inability to quench it. The origin of the resting-state radical itself from an interrupted and thus unproductive catalytic cycle may serve as a protective mechanism.

So far, we do not know the physiological substrates of B-type DyPs. Their structural features most probably are completely different compared to those of ABTS or other bulky model substrates. On one hand, Compound I in all B-type *Kp*DyPs is efficiently and rapidly formed by H_2_O_2_ and remarkably stable.^11,29−31,33,79^ On the other hand, we could demonstrate the presence of a LRET path from Y247 to the heme periphery. Nevertheless, a catalytically active Compound I* [oxoiron(IV) Y243 radical] could never be formed upon addition of hydrogen peroxide. This suggests that Compound I* formation may be triggered through substrate-induced structural changes that promote LRET. This might provide a useful valve that inhibits the formation of surface-exposed oxidation sites and oxidative damage in the absence of the physiological electron donor. Whether Y247 is indeed a biologically relevant oxidation site can be determined only once the biological substrate is identified.

## Supporting Information Available

X-Band CW EPR spectra at 10 K of WT *Kp*DyP and *E. coli* YfeX (*Ec*DyP) (Figure S1) and X-band HYSCORE spectra of WT *Kp*DyP (Figure S2), UV–vis spectra of investigated variants (Figure S3), and first derivative of W-band ESE-detected EPR spectra of the radical species in *Kp*DyP variants Y82F, Y237F, and W176F (Figure S4) (PDF)

## Accession Codes

*Kp*DyP, A0A0W8ATM9; C*c*P, P00431; *Pc*LiP, Q01787; *Tce*LiP, Q3C1R8; *Rj*DyP, Q0S4I5; *El*DyP, E3G9I4; *Pp*DyP, A0A126S5E0; *Aau*DyP, I2DBY1; EfeB, P31545; *Sl*DyP, A0A076LSE4; *Tc*DyP, D1A807; *Ec*DyP, P76536; *Vc*DyP, A0A085T8S6; *Synechocystis* PCC6803 KatG, P73911; *Mt*KatG, P9WIE5; turnip isoperoxidase 7, P00434.
