# Supplementary material for: On the Track of Long-Range Electron Transfer in B-Type Dye-Decolorizing Peroxidases: Identification of a Tyrosyl Radical by Computational Prediction and Electron Paramagnetic Resonance Spectroscopy
Source: Biochemistry. 2021 Mar 30;60(15):1226–41. doi: 10.1021/acs.biochem.1c00129 (PMC8154254; doi:10.1021/acs.biochem.1c00129)
Supplement: Supplementary file 1 — bi1c00129_si_001.pdf [file bi1c00129_si_001.pdf]

Supporting Information to

On the track of long-range electron transfer in B-type dye-decolorizing peroxidases – Identification of a tyrosyl radical by computational prediction and EPR spectroscopy

Kevin Nys[1], *Paul. G. Furtmüller*[2], *Christian Obinger*[2], *Sabine Van Doorslaer**[1], *Vera Pfanzagl**[2]

[1] BIMEF Laboratory, Department of Chemistry, University of Antwerp, 2610 Antwerp, Belgium

[2] Department of Chemistry, Institute of Biochemistry, BOKU - University of Natural Resources and Life Sciences, 1190 Vienna, Austria

*to whom correspondence should be addressed:

Vera Pfanzagl: email vera.pfanzagl@boku.ac.at, telephone +43 1 47654-77278

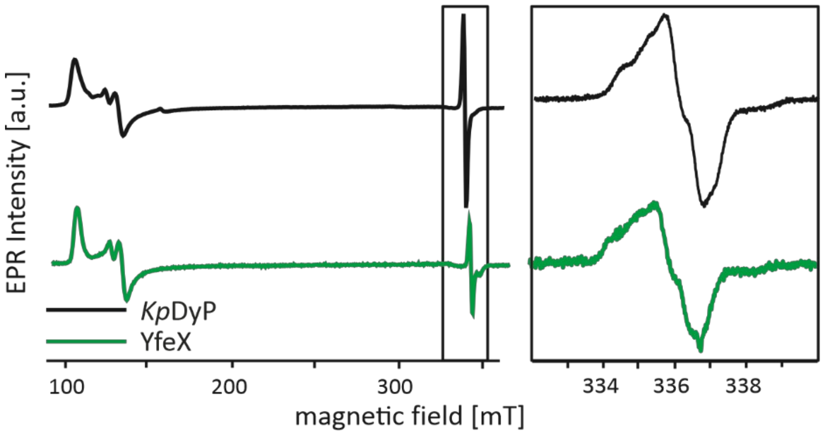

Figure S1: right: X-band CW EPR at 10 K of WT *Kp*DyP and *E. coli* YfeX (*Ec*DyP) and left: a zoom of the radical species at 80K in the resting-state enzymes

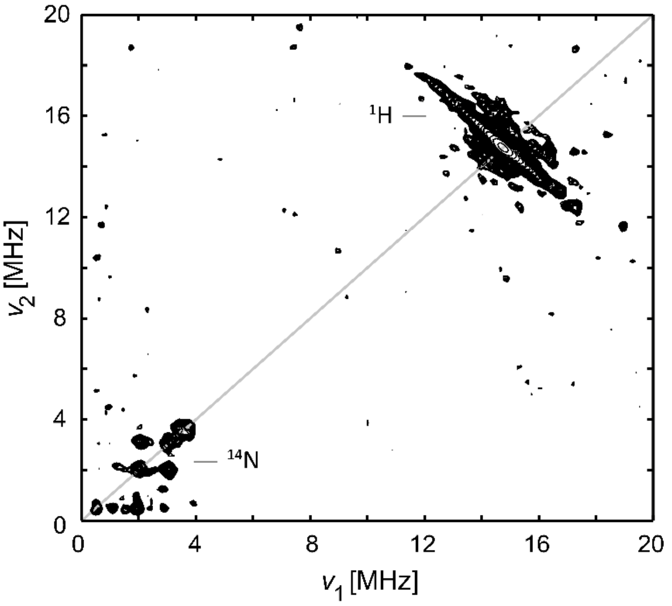

Figure S2: X-band HYSCORE of WT *Kp*DyP at 40 K, at a magnetic field setting corresponding with $g \approx 2.005$ and averaged over two τ-values (104 ns and 184 ns). Cross peaks centred around 2.5 MHz reveal the weak hyperfine coupling of a ^{14}N nucleus. A ridge at 14.7 MHz is due to proton hyperfine couplings.

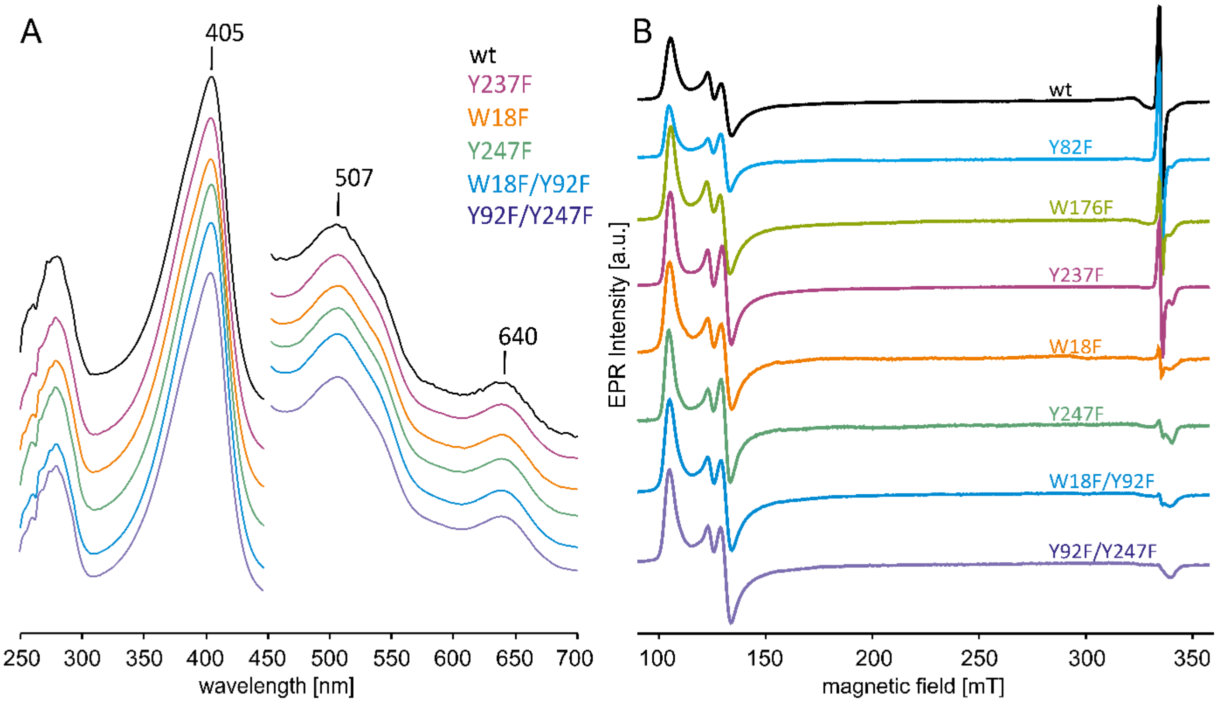

Figure S3: (A) Room-temperature UV-vis spectra of WT *Kp*DyP and selected variants and (B) X-band CW EPR spectra of WT *Kp*DyP and all variants at 10 K.

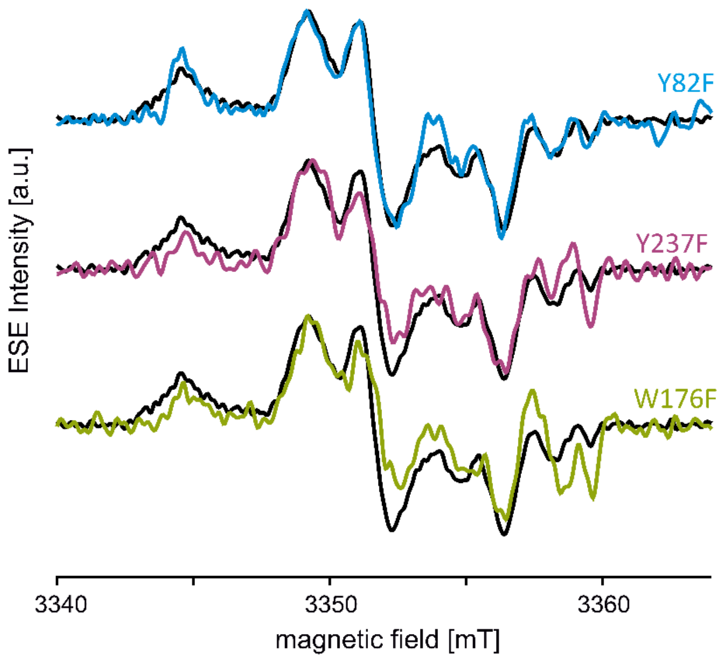

Figure S4: 1st derivative of W-band ESE-detected EPR (94 GHz, τ = 340, 400 ns) of the radical species in the *Kp*DyP variants Y82F (light blue), Y237F (magenta) and W176F (olive) in comparison with the wild-type (black).
